# Non-secretory multiple myeloma with unusual TFG-ALK fusion showed dramatic response to ALK inhibition

**DOI:** 10.1038/s41525-021-00186-9

**Published:** 2021-03-17

**Authors:** Ashiq Masood, Trevor Christ, Samia Asif, Priya Rajakumar, Beth A. Gustafson, Leyla O. Shune, Ameen Salahudeen, Drew Nedvad, Suparna Nanua, Agne Paner, Timothy M. Kuzel, Mia Levy, Janakiraman Subramanian, Shahzad Raza

**Affiliations:** 1 Division of Hematology/Oncology and Cell Therapy, Rush University Medical Center, Chicago, IL 60612 USA; 2 Rush Precision Oncology Program, Rush University Medical Center, Chicago, IL 60612 USA; 3 Department of Pharmacy, Rush University Medical Center, Chicago, IL 60612 USA; 4 Department of Medicine, University of Missouri-Kansas City School of Medicine, Kansas City, MO 12 64108 USA; 5 Precision Oncology Program, St Luke’s Cancer Institute, Kansas City, MO 64111 USA; 6 Department of Hematologic Malignancies and Cellular Therapeutics, University of Kansas Medical Center, Kansas, KS 66205 USA; 7 Tempus Labs, Inc, Chicago, IL USA; 8 MAWD Pathology Group, North Kansas City, MO 64116 USA; 9 Department of Pathology, St Luke’s Cancer institute, Kansas City, MO 64111 USA; 10 Division of Oncology, Saint Luke’s Cancer Institute, University of Missouri School of Medicine, Kansas City, MO 64111 USA

**Keywords:** Molecular medicine, Cancer genomics

## Abstract

Non-secretory multiple myeloma (NSMM) constitutes a distinct entity of multiple myeloma characterized by the absence of detectable monoclonal protein and rarely an absence of free light chains in the serum and urine. Given its rarity, the genomic landscape, clinical course, and prognosis of NSSM are not well characterized. Here, we report a case of a patient with relapsed and refractory NSMM with brain metastasis harboring a *TFG-ALK* fusion showing a dramatic and durable (over two years) response to commercially available anaplastic lymphoma kinase (ALK) inhibitors. The case emphasizes the beneficial role of molecular profiling in this target-poor disease.

## Introduction

Non–secretory multiple myeloma (NSMM) is a rare variant of multiple myeloma (MM) and constitutes 3% of total MM cases^1^. The underlying pathology of NSMM is reported to be either failure of malignant plasma cells to form immunoglobulin (non-producer) or a failure to secrete heavy or light chains (non-secretor)^2^. Pathogenesis in MM is governed by the acquisition of multiple somatic sub-clonal secondary genomic events, including copy number abnormalities, secondary translocations, acquired genomic and epigenetic mutations, and DNA hypomethylation^3,4^. Subclonal events include deletion of *RB1*, *TP53*, *PTEN*, amplification of chromosome 1q, activating point mutations in *KRAS*, *NRAS,* and *BRAF*, though they can be clonal and may occur at any point in the disease course^5,6^, A recent study by Morgan et al.^6^, has shown low frequency (2.7%, *n* = 958) of non-immunoglobulin fusions in MM patients. These fusion genes were present in smoldering MM, newly diagnosed MM, and relapsed MM patients, indicating these fusion genes occur early in the disease process. Fifty-four percent of these fusion genes were in-frame fusions, potentially resulting in functional oncogenic fusion proteins. Most of these in-frame tyrosine kinase fusions were sub-clonal and therefore, secondary events. The exception is the EML4-ALK fusion gene, which was clonal and thus, therapeutically targetable with anaplastic lymphoma kinase (ALK) inhibitors. *ALK* fusion partners are identified as driver events in nearly twenty different human malignancies, including non-small cell lung cancers (NSCLC), anaplastic non-Hodgkin lymphoma, diffuse large B cell lymphoma, renal carcinoma, thyroid cancers, breast cancer, ovarian carcinoma, leukemia, and MM^6–8^. These fusions lead to constitutive activation of ALK kinase which subsequently induces the activation of downstream pathways, including PI3K/protein kinase B and the extracellular signal-regulated kinase (ERK)/MAPK pathway^9^.

In the rapidly evolving field of MM, we have seen a remarkable reduction in mortality rates in clinical trials and population-based studies. Unfortunately, patients with NSMM are frequently excluded from clinical trials. Furthermore, molecular studies in NSMM are lacking. Here, we report a case of patient with NSMM harboring a trafficking from ER to Golgi regulator gene (*TFG*, formerly TRK fused gene) and ALK fusion *(TFG-ALK fusion*), which demonstrated a dramatic response to ALK inhibition.

## Results

### Case

A 49-year-old Caucasian gentleman initially underwent surgical resection of an 8.7 cm transmural jejunal mass. Pathology was consistent with a kappa-restricted plasmacytoma with multiple negative adjacent lymph nodes. He had a normal renal function, calcium, complete blood count, serum and urine immunofixation electrophoresis as follows: serum-free light chains, free Kappa 14.7 mg/L (Normal value: 3.3–19.4 mg/L), free Lambda 14.5 mg/L (Normal value: 3.3–19.4 mg/L) and Kappa/Lambda ratio 1.01 (Normal value: 0.26–1.65). The skeletal survey and positron emission tomography (PET)/CT did not show lytic lesions. Bone marrow biopsy was negative for clonal plasma cells. The patient was closely followed by imaging surveillance for an isolated plasmacytoma.

Three years later, he presented with right cervical lymphadenopathy and a new nasopharyngeal mass. Lymph node biopsy was consistent with kappa-restricted plasmacytoma. Repeat paraproteinemia workup was consistent with NSMM. Re-staging PET/CT showed multiple abnormal radiotracer uptake areas, including the nasopharyngeal mass (5.4 × 4.9 × 4.0 cm, SUV 18.4), necrotic right cervical lymph node, a focal area in the jejunum, right adrenal gland, and soft-tissue chest nodules. He was treated with KRd (carfilzomib, lenalidomide, dexamethasone). His tumor initially responded to the KRd regimen with a complete metabolic response on imaging, but after six cycles of KRd, the tumor recurred with a small bowel mass with an SUV of 14.1 on PET/CT. Unfortunately, he continued to progress despite changing therapies including, KCd (carfilzomib, cyclophosphamide, dexamethasone), local radiation to the jejunal mass, and the DPd regimen (daratumumab, pomalidomide, dexamethasone). Within two months after starting the DPd regimen, he developed diffuse hepatic lesions and a loculated pelvic mass (Supplementary Fig. 1A, B). Liver biopsy showed kappa restricted plasma cells, consistent with plasmacytoma (Supplementary Fig. 2A–D). These plasma cells are characterized by increased nuclear to cytoplasm ratio, variable dispersed chromatin, prominent nucleoli, and modest eosinophilic cytoplasm. Broad immunohistochemistry panel was performed including CD3, CD5, CD19,CD20, CD33, CD34, LCA, CD56, CD68, CD79a, CD117, CD138 CAM5.2, myeloperoxidase, pankeratin, Cyclin D1, IgA, IgD, IgG, IgM, lysozyme, MUM-1, PAX5, ALK-1, HHV8, EBV, and kappa and lambda. The neoplastic plasma cells showed a diffuse proliferation of CD138+ plasma cells. In situ hybridization for kappa and lambda light chains demonstrates a kappa restricted plasma cell population. CD19 and CD20 were negative on neoplastic plasma cells. CD43, LCA, and CD117 were partially positive. Additional testing including *BRAF* mutation by PCR and ALK-1 stain were negative. Multiple pathology consultations were obtained at different institutes, and the consensus opinion was that findings were consistent with plasmacytoma. Fluorescent in-situ hybridization (FISH) showed 17p13.1 and trisomy 7 in 63% and 60% plasma cells, respectively. The patient received two cycles of salvage chemotherapy with VPD-PACE (bortezomib, pomalidomide, dexamethasone, cisplatin, doxorubicin, cyclophosphamide, and etoposide) with complete metabolic response followed by autologous stem cell transplant (ASCT). Post-ASCT, he started maintenance therapy with the VPd regimen (bortezomib, pomalidomide, and dexamethasone). However, within six months, he developed multiple new foci of PET FDG uptake involving the thoracic spine, mediastinum, and the abdomen.

After obtaining informed consent, we obtained a liver biopsy and collected peripheral blood for a commercially available CLIA certified genomic assay. Whole-genome sequencing (WGS) and RNA sequencing were performed using the commercially available NantHealth platform. A total of 591 somatic variants were identified in this patient’s tumor, including 147 non-synonymous variants, for an estimated exonic mutation rate of 5.8 mutations per megabase (Mb), suggesting a low tumor mutation burden (TMB). Among the somatic variants identified, three variants were considered to be pathogenic (1 nonsense mutation in *MYO6* and two frame-shift mutations in *TP53* and *TNFAIP3*). Two variants were considered likely pathogenic (*HGF* and *GRID1*), both of which were missense mutations. Five hundred forty-nine genes were considered benign or likely benign, with 37 variants currently of unknown significance. Details of pathogenic alterations are detailed in Table 1. No actionable mutations were identified based on whole-genome sequencing. RNA sequencing to detect the presence of possible fusions between two transcripts (with one of the transcripts belonging to one of 74 genes commonly found in oncogenic fusions) was assessed. Reportable fusions must both be classified as functional and have greater than eight reads supporting the fusion junction was considered significant. Based on this analysis, one fusion transcript was identified: TFG-ALK, with protein description as t(3;2)(TFG:p.M1_N138; ALK:p.V1058_*1621), and exon composition as *TFG*(e1-4) + *ALK*(e20-29) (Table 1). The case was presented in Molecular Tumor Board and *ALK* inhibitor therapy was discussed.

**Table 1:** Genomic characteristics of TFG-ALK fusion and pathologic gene signature of liver biopsy.

Fusion	TFG-ALK			
Protein discription	(3;2) (*TFG*p.M1_n138; *ALK*:p.V1058_*1621)			
Exon composition	TFG(e1-4) + ALK(e20-29)			
TFG RNA expression	62.2 TPM^*^			
ALK RNA expression	13.0 TPM^*^			
TFG-ALK Fusion Support	154 reads			
Gene	Pathogenicity	Mutation Type	DNA VAF^^^ (%)	RNA VAF^^^ (%)
*TP53* p.H178Tfs*69	Pathogenic	Frameshift	45.0	36.4
*TNFAIP3* p.C627Ffs*44	Pathogenic	Frameshift	25.0	1.9
*MYO6* p.E953*	Pathogenic	Nonsense	28.2	11.1
*HGF* p. G674D	Likely Pathogenic	Missense	37.6	1.4
*GRID1* p.S556Y	Likely Pathogenic	Missense	27.5	0.0

^*^TPM transcripts per kilobase Million, ^^^*VAF* Varient Allele Frequency.

**Fig. 1:**
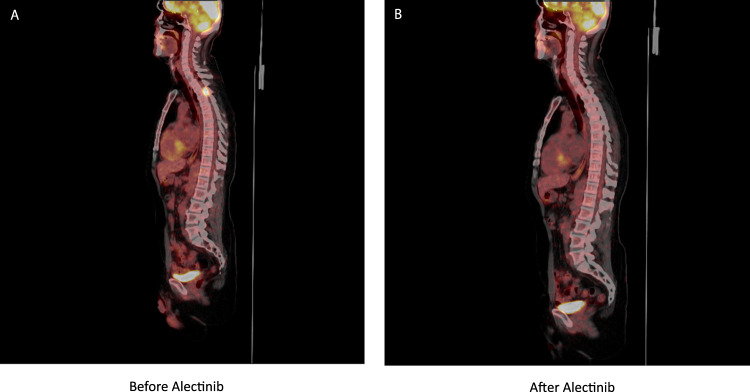
PET Scan results. **A** Before initiation of Alectinib. Foci of increased uptake in a mediastinal lymph node (max SUV 6.3), retrocaval lymph node (max SUV 10.6), right T3/T4 intervertebral foramen (max SUV 14) on PET-CT. **B** Complete response after 2 months of alectinib. Complete resolution of prior noted upper thoracic spine lesion and upper abdominal pericaval nodule.

**Fig. 2:**
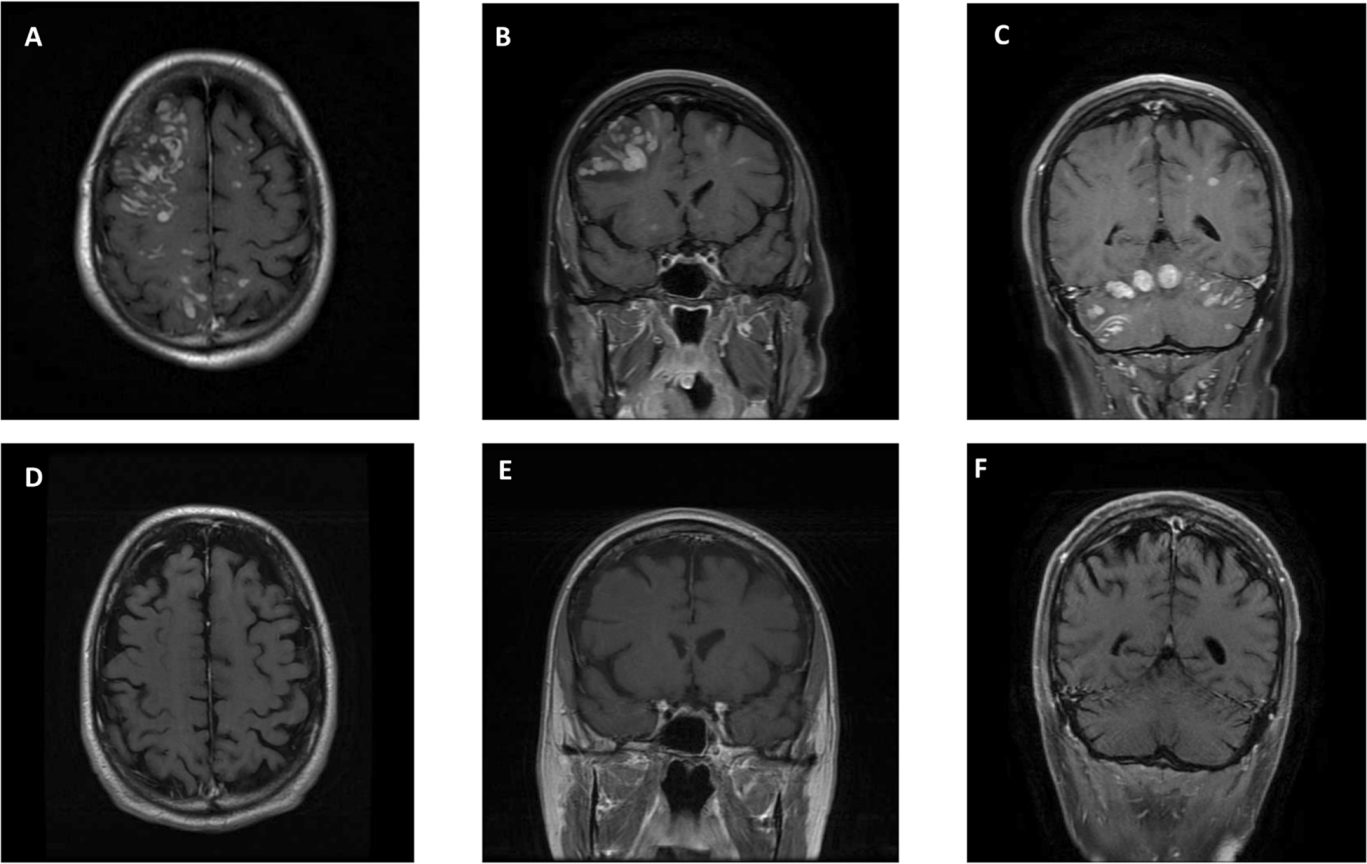
MRI Brain before and after Loralatinib treatment. **A**, **B**, **C** MRI of the brain before starting Lorlatinib (pre-treatment) showing innumerable diffuse enhancing lesions with surrounding vasogenic edema, with a right frontal lobe and bilateral cerebellar predominance. **D**, **E**, **F** Treatment response to lorlatinib with markedly decrease in size and number of brain metastasis and markedly improved cerebral edema.

Since this particular *TFG-ALK* t(3;2) fusion had structural similarity to other well-known *ALK* fusion variants, it was expected to be targetable by an ALK inhibitor. The patient was initiated on compassionate treatment with the second-generation ALK inhibitor, alectinib 600 mg twice daily. Repeat PET/CT imaging after two months showed resolution of the thoracic lesion and abdominal lymph nodes (Fig. 1). After two months on alectinib, the patient did not have access to further treatment and unfortunately demonstrated relapsed disease in the brain involving multiple innumerable diffuse enhancing lesions with surrounding vasogenic edema involving the right frontal lobe and bilateral cerebellar predominance (Fig. 2). Cerebrospinal fluid (CSF) analysis showed evidence of plasmablasts (not seen on his previous CSF analysis) (Supplementary Fig. 3). He was treated with whole-brain radiation therapy, intrathecal methotrexate, and started on lorlatinib, a 3rd generation ALK inhibitor at 100 mg orally daily. At the time of writing this report, the patient has been maintained for >2 years on lorlatinib. His brain MRI and PET/CT are negative for recurrence (Figs. 1 and 2), demonstrating a durable response to targeted ALK inhibition surpassing conventional systemic myeloma therapy including autologous stem cell transplantation.

## Discussion

**Fig. 3:**
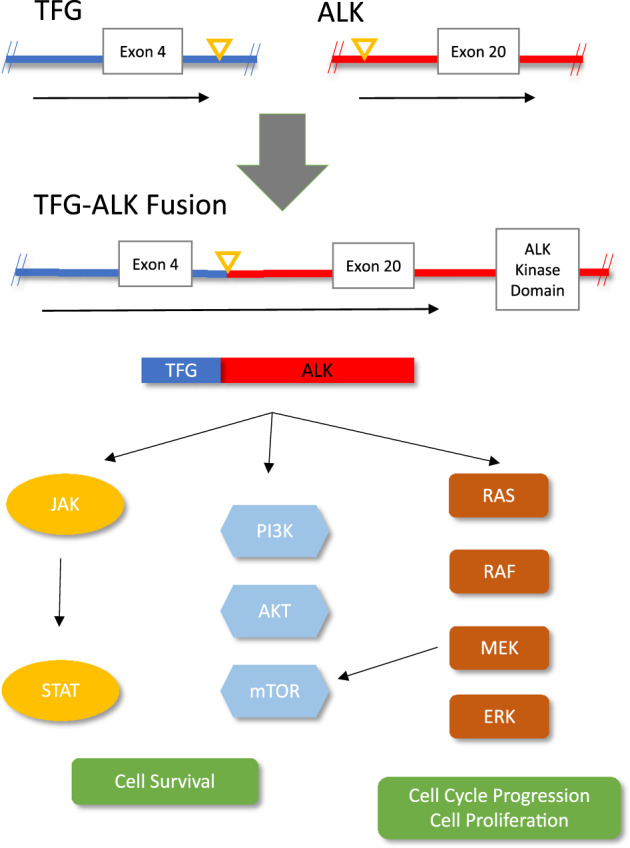
TFG-ALK fusion and downstream pathway activation. TFG-ALK fusion product is displayed as a t(3;2) fusion at exon 4 of *TFG* (blue) and exon 20 of *ALK* (red). The point of the fusion is indicated by the yellow triangle. The druggable ALK kinase domain resides downstream of ALK exon 20. The upregulated ALK pathway by means of fusion product is implicated in many oncogenic cellular pathways including JAK/STAT, PI3K/mTOR, and RAS/RAF.

This is a unique case demonstrating significant clinical response using commercially available ALK inhibitors in a *TFG-ALK* fusion in relapsed/refractory NSMM. The *ALK* gene encodes the *ALK* tyrosine-kinase protein, a receptor tyrosine kinase, known to have vital roles in normal physiologic processes, including the transmission of extracellular signals implicated in proliferation, differentiation, and survival (Fig. 3). Plasma cell neoplasms are distinct hematologic neoplasms where the diagnosis is based on morphology, immunohistochemistry, FISH, and conventional karyotyping. Case studies have previously reported *ALK* fusion partners *EML4-ALK* and *CLTC-ALK* in these patients^6,10^.

The *TFG* was previously identified as a fusion partner for *ALK* in cases of Anaplastic large cell lymphoma (ALCL) t(2;3) (p23;q21). The *ALK* breakpoint in these translocations was the same as in the classical t(2:5). The chimeric protein contains cytoplasmic domain ALK and coiled-coil domain of *TFG*. The fusion protein was thus able to dimerize, resulting in constitutive kinase activity^11^. Alectinib is currently Food and Drug Administration (FDA) approved for ALK-positive metastatic NSCLC. It is an orally bioavailable TKI that inhibits ALK and RET proteins by preventing their phosphorylation. The inhibition of activation of ALK impairs downstream signaling of cell proliferation^12^. Lorlatinib is approved in NSCLC carrying ALK fusion and has shown superior intracranial activity even in patients treated with other ALK inhibitors^13^. Therefore, it was an ideal choice for our patient after CNS metastasis development.

Our case highlights that a small percentage of patients with plasma cell neoplasms carries ALK fusion and may benefit from ALK inhibition. The diagnosis of ALK+ NSMM versus large B cell lymphoma is challenging because of its rarity, unique morphologic characteristics, and unusual immunophenotypic features, which significantly overlap with other hematologic and nonhematologic neoplasms. Little is known about the clinical behavior of NSMM. We acknowledge that the clinical behavior of NSMM may resemble plasmablastic lymphoma. Due to these concerns, we have obtained multiple opinions on pathology and there is a uniform consensus on the diagnosis of plasmacytomas (NSMM). In addition, ALK-1 stain and EBV stain were absent on plasmacytomas. We believe 17p deletion, which is considered a high-risk mutation in these patients, is most likely the cause of aggressive clinical behavior in our case. This case demonstrates the potential role of next-generation sequencing (NGS) in NSMM, a disease where NGS is not currently standard of care and morphological or immunophenotypical features define diagnosis and influence treatment decisions. NGS may identify unrecognized targetable oncogenic drivers such as ALK-fusions. Detection of these gene alterations not only improves our understanding of disease pathogenesis but may also be used to exploit the therapeutic vulnerabilities of these tumors. The therapeutic success seen in this case emphasizes the need to identify such molecular targets and unique driver alterations, especially in the relapsed setting with a disease that was refractory to traditional treatment options. Our knowledge of mechanisms involved in MM, especially NSMM oncogenesis, is incomplete and further molecular studies are warranted to better understand biological underpinnings that may improve outcomes in these patients. After failing more than five lines of systemic treatment, our patient is currently in clinical and radiographic remission with ALK inhibitor over two years. Our case is a successful example of utilizing NGS-based mutational profiling and incorporating unconventional targeted agents into clinical practice in a rare disease.

## Methods

The patient provided written informed consent for carrying out Next-Generation Sequencing and publication of case reports using CLIA certified commercially available Nant platform, as previously described^14^. The analysis is also described in the Nant GPS report. Briefly, DNA libraries were prepared using the KAPA Hyper prep kit and sequencing on Illumina sequencing platform. DNA sequencing data was aligned to Genome Reference Consortium Human Build 37 using Burrows-Wheeler Aligner^15^. The germline, somatic variant detection, insertion, and deletions were carried out using the NantOmics Contraster pipeline^16–18^. RNA-seq libraries were prepared using KAPA Stranded RNA-Seq with RiboErase kit and sequenced on the Illumina Sequencing platform. RNA sequencing data was aligned used bowtie2^19^. RNA transcript expression was assessed by RSEM^20^. RNA fusions are detected using transcriptome-aligned RNA sequencing data. When there is evidence of gene-fusion using clusters of spanning reads between two transcripts, the Nant team performs de novo assembly on all sequencing data to detect the precise location of fusion transcript. Only fusion with open reading frames that extend to the downstream partner’s stop codon is reported.

The patient was treated with lorlatinib and alectinib within an individual program after informed consent. IRB approval (St. Luke’s Hospital, Kansas City, MO) was waived by the IRB.

### Imaging

PET-CT and MRI imaging were obtained as standard of care.

### Reporting summary

Further information on research design is available in the Nature Research Reporting Summary linked to this article.

## Supplementary information

Supplementary Information

Reporting Summary

## Data Availability

The authors declare that data supporting the findings of this study are available within the paper. RAW data files Including FASTQ and BAM files are the property of commercial platform NANTOMICS and authors do not have access to these files. The de-identified HIPAA compliant clinical Genomics report of the current study is available.
