# Supplementary figures and images for: Non-secretory multiple myeloma with unusual TFG-ALK fusion showed dramatic response to ALK inhibition
Source: NPJ Genom Med. 2021 Mar 17;6:23. doi: 10.1038/s41525-021-00186-9 (PMC7969947; doi:10.1038/s41525-021-00186-9)

Supplementary Figure 1 A-B

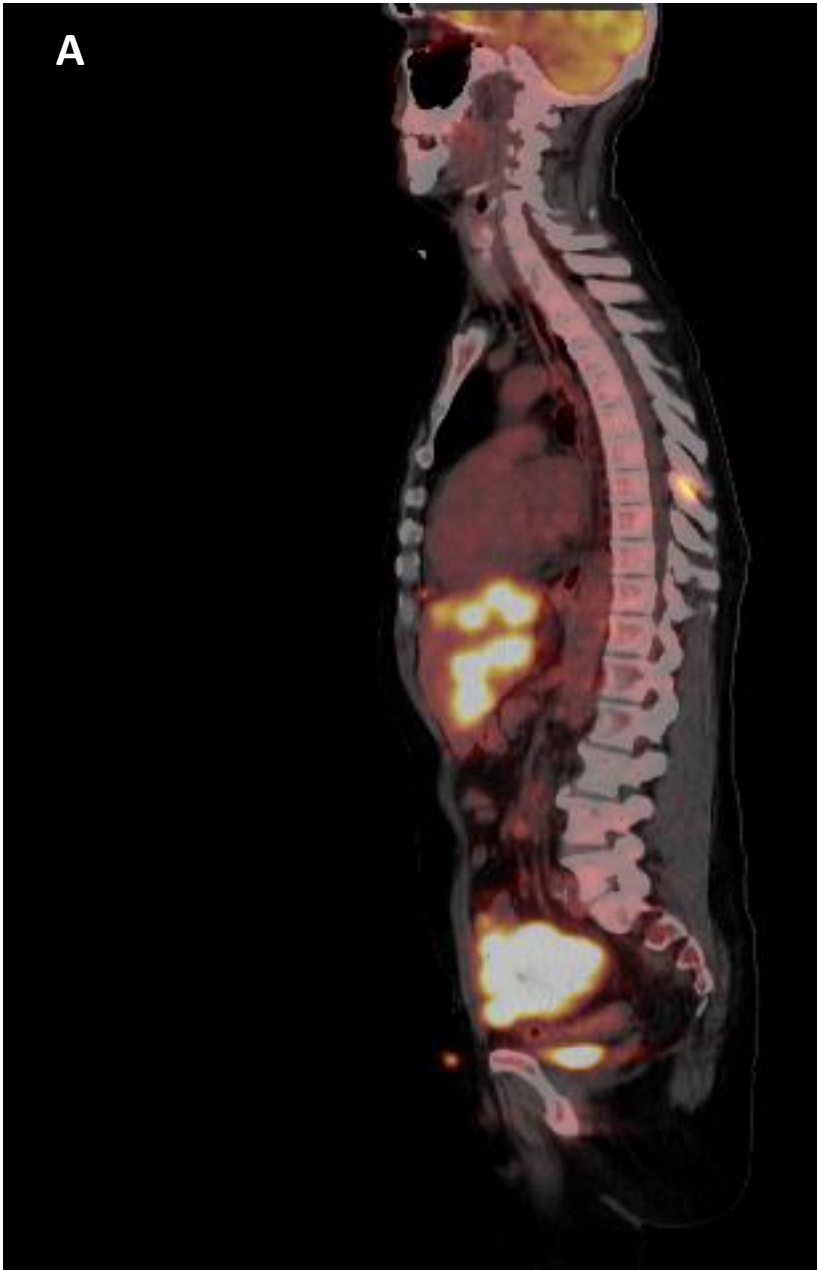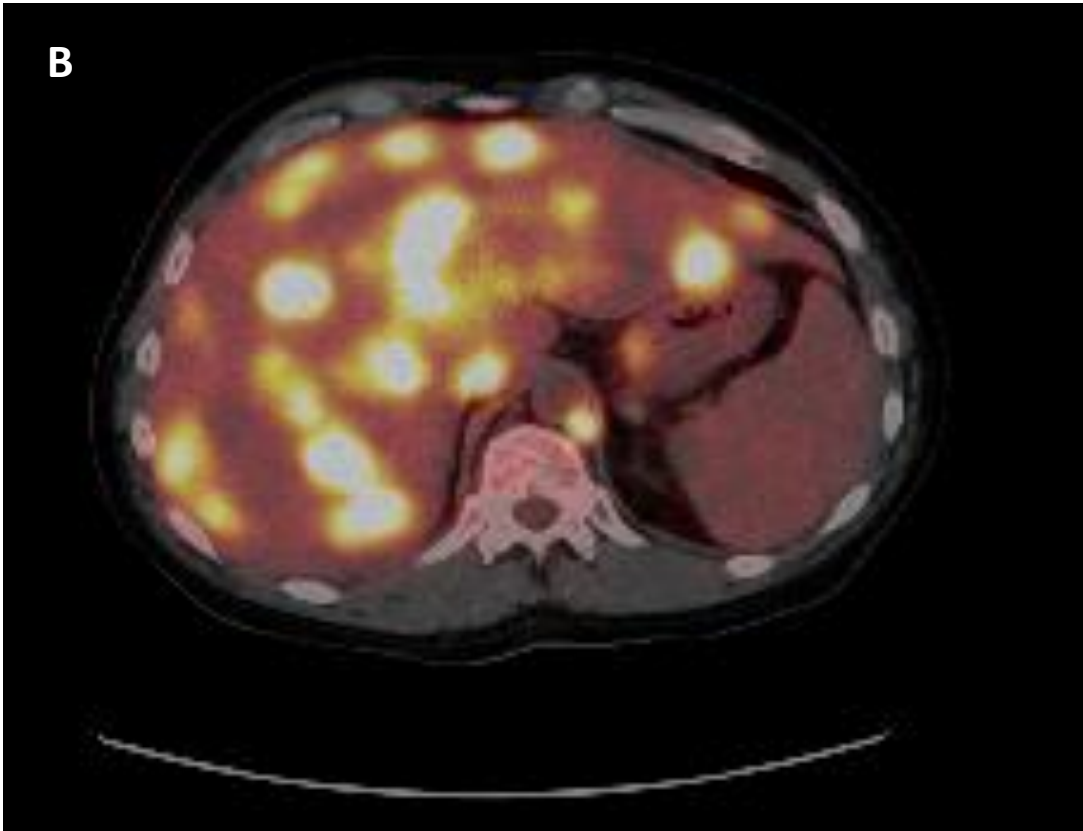

Supplementary Figure 2A-D

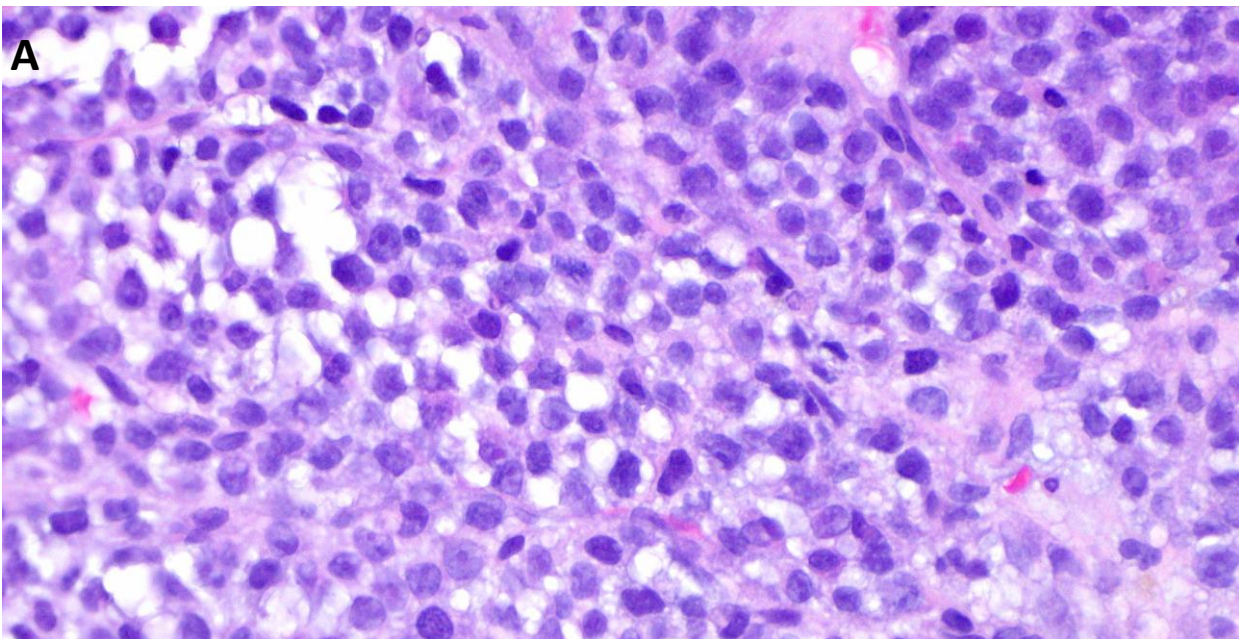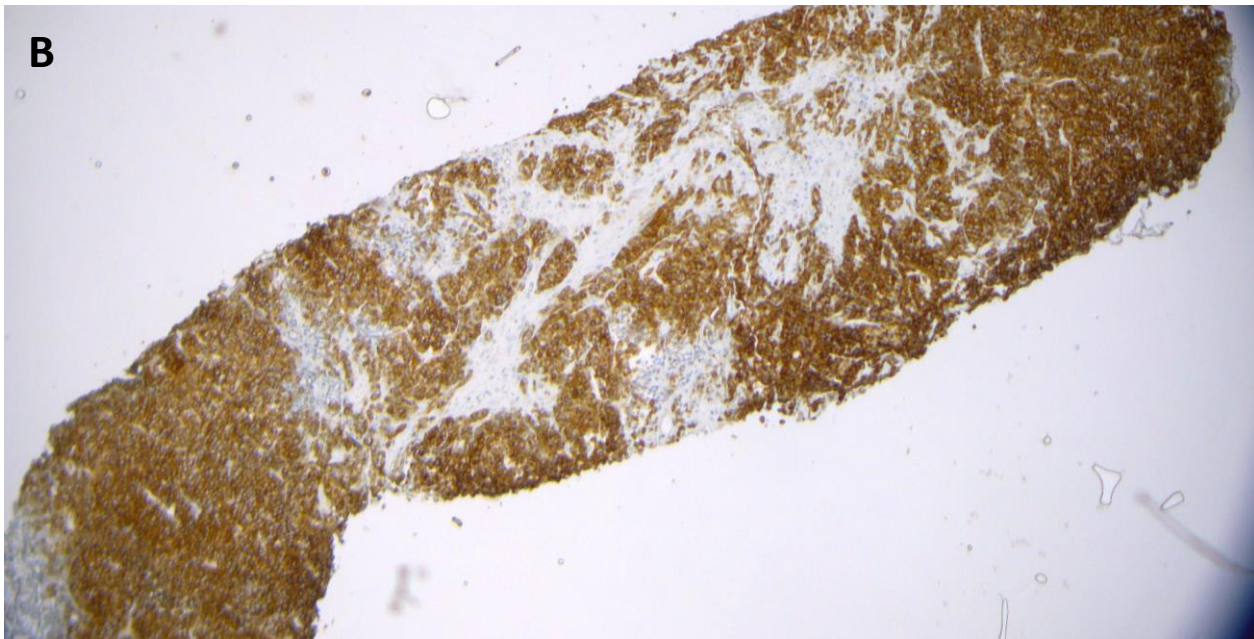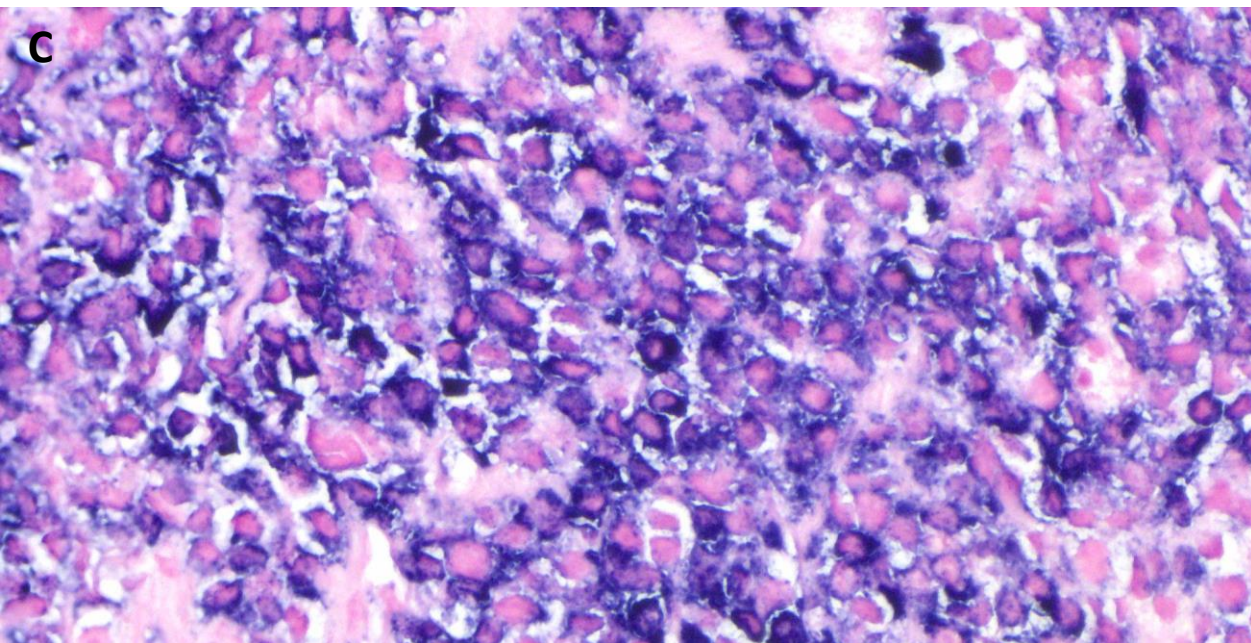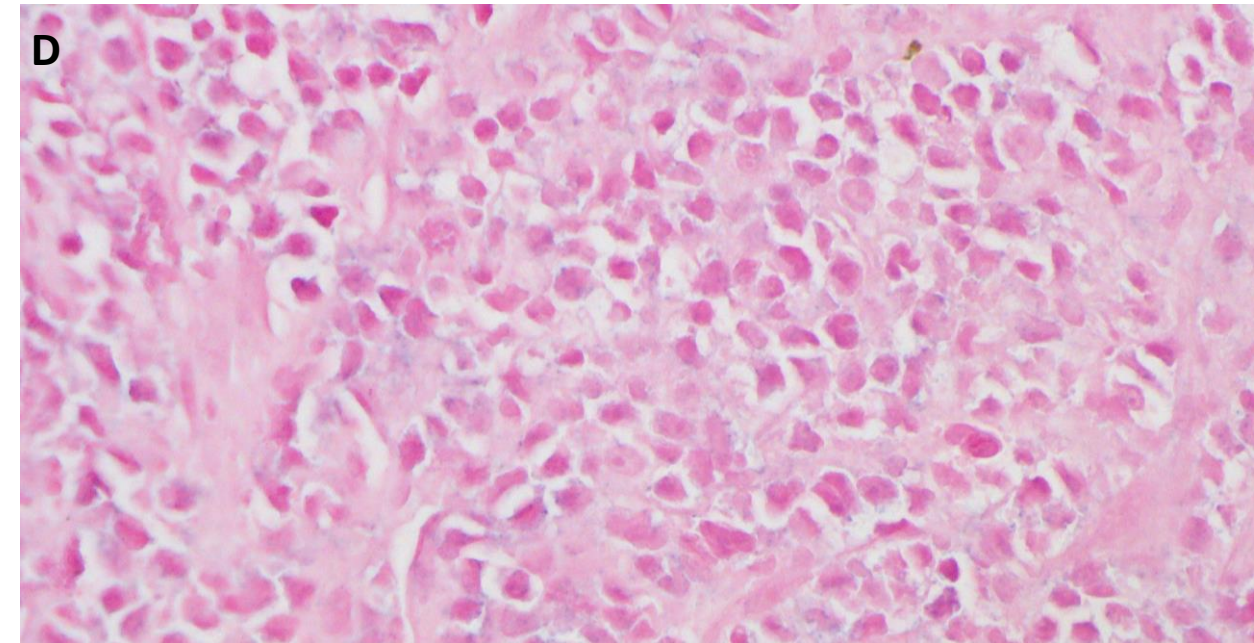

Supplementary Figure 3

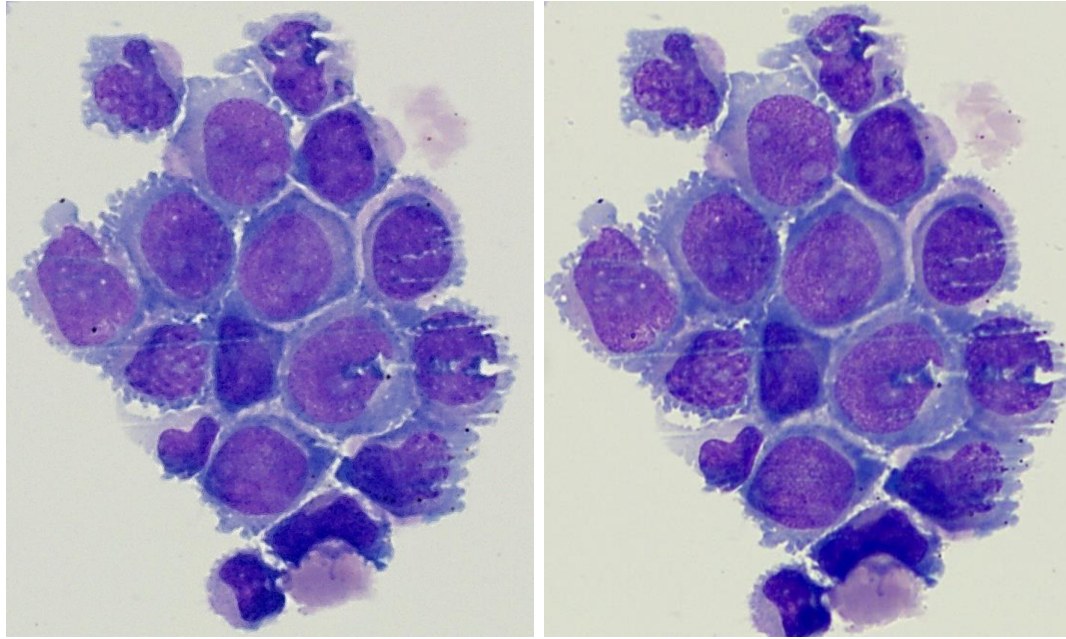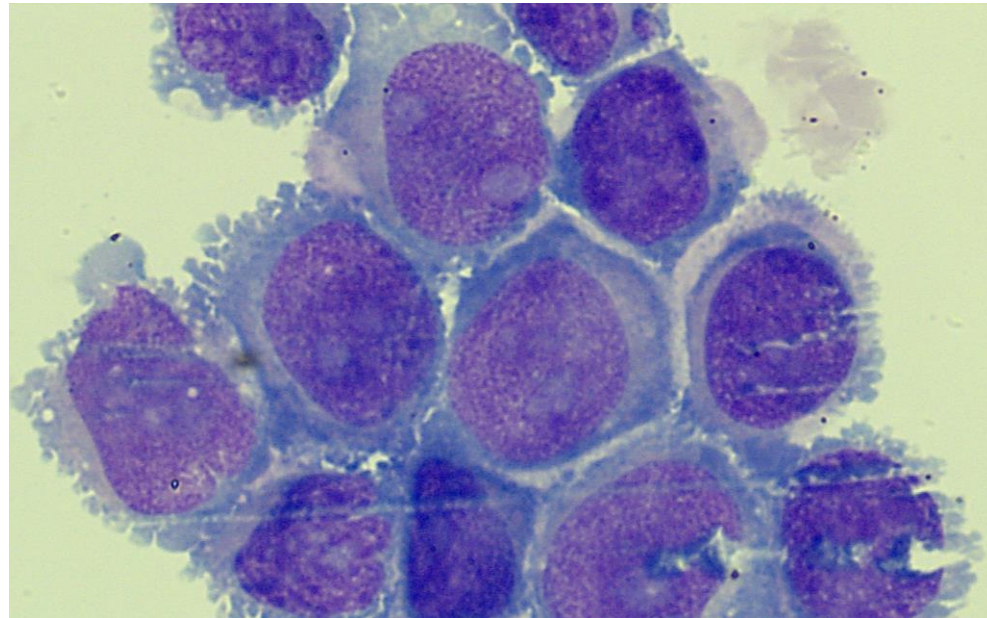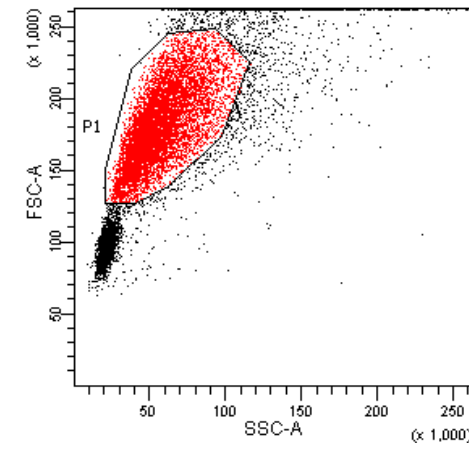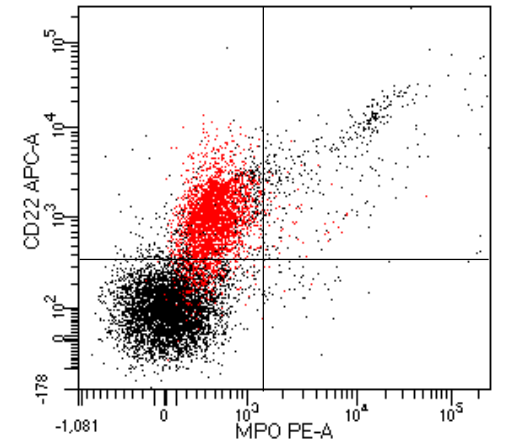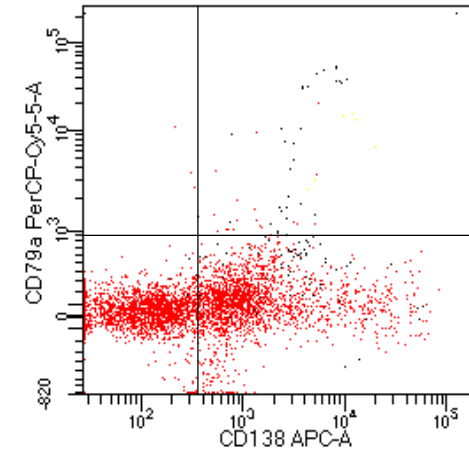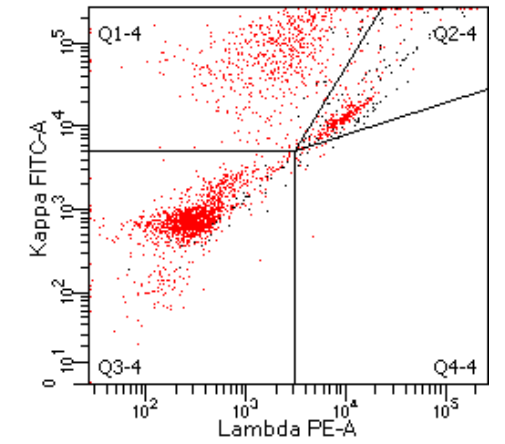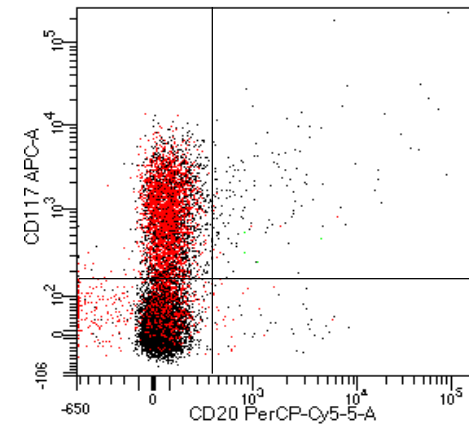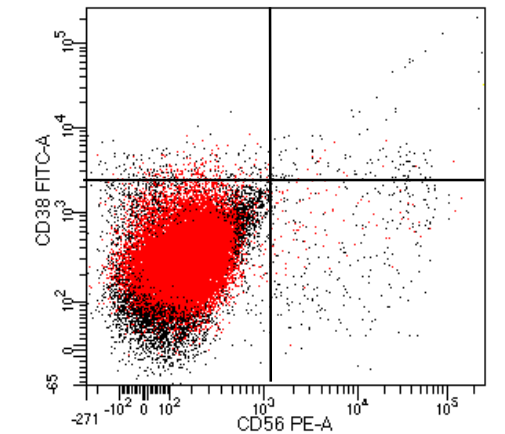

Supplement: Supplementary file 1 — Supplementary Information [file 41525_2021_186_MOESM1_ESM.pdf]
